# The complete mitochondrial genome of stag beetle *Lucanus cervus* (Coleoptera: Lucanidae) and phylogenetic analysis

**DOI:** 10.7717/peerj.8274

**Published:** 2019-12-19

**Authors:** Dan Chen, Jing Liu, Luca Bartolozzi, Xia Wan

**Affiliations:** 1 School of Resources and Environmental Engineering, Anhui University, Hefei, Anhui, China; 2 Department of Entomology, Natural History Museum of the University of Florence, Zoological Section “La Specola”, Natural History Museum of the University of Florence, Florence, Italy

**Keywords:** Bayesian inference analysis, Phylogenetic analysis, Mitogenome, The next generation sequencing, *Lucanus cervus*

## Abstract

**Background:**

The stag beetle *Lucanus cervus* (Coleoptera: Lucanidae) is widely distributed in Europe. Habitat loss and fragmentation has led to significant reductions in numbers of this species. In this study, we sequenced the complete mitochondrial genome of *L. cervus* and reconstructed phylogenetic relationships among Lucanidae using complete mitochondrial genome sequences.

**Methods:**

Raw data sequences were generated by the next generation sequencing using Illumina platform from genomic DNA of *L. cervus*. The mitochondrial genome was assembled by IDBA and annotated by MITOS. The aligned sequences of mitochondrial genes were partitioned using PartitionFinder 2. Phylogenetic relationships among 19 stag beetle species were constructed using Maximum Likelihood (ML) method implemented in IQ-TREE web server and Bayesian method implemented in PhyloBayes MPI 1.5a. Three scarab beetles were used as outgroups.

**Results:**

The complete mitochondrial genome of *L. cervus* is 20,109 bp in length, comprising 13 protein-coding genes, 22 transfer RNA genes, two ribosomal RNAs and a control region. The *A* + *T* content is 69.93% for the majority strand. All protein-coding genes start with the typical ATN initiation codons except for *cox1*, which uses AAT. Phylogenetic analyses based on ML and Bayesian methods shown consistent topologies among Lucanidae.

## Introduction

Many species from the family of Lucanidae (stag beetles) are facing conservation concerns in many regions of the world. Some stag beetles are used as flagship species to raise public awareness of their protection (New, 2018). Understanding the phylogenetic relationships among species of Lucanidae will be helpful to reveal the evolutionary history of this special group and conduct conservation in practice. Previous studies have tried to construct the phylogenetic relationships among Lucanidae using partial mitochondrial and nuclear genes (Hosoya & Araya, 2005; Kim & Farrell, 2015). With the development of next-generation sequencing, it is possible to get more molecular markers for phylogenetic analysis to get more robust relationships (Fagua et al., 2018; Li et al., 2015).

The mitochondrial genome is a double-stranded circular molecule. Due to its features of material inheritance, stability of gene content and easy sequencing, it has been widely used in phylogenetic inference in many groups of animal from higher levels to lower levels (Li et al., 2016; Zheng et al., 2018). Currently, 20 mitochondrial genomes were used from the family of Lucanidae, representing four tribes, 14 genera (Table 1). Compared to the number of known species in Lucanidae, the sequenced mitochondrial genomes are still limited.

**Table 1:** GenBank accession numbers of species used in this study.

Family	Species	GenBank Accession number	References
Lucanidae	*Cyclommatus vitalisi*	MF037205	Liu et al. (2017)
	*Dorcus curvidens hopei*	MF612067	Chen et al. (2018)
	*Dorcus hansi*	MF621709	Direct submission
	*Dorcus parallelipipedus*	KT876887	Linard et al. (2016)
	*Lucanus cervus*	MN580549	This study
	*Lucanus fortunei*	MF614013	Direct submission
	*Lucanus mazama*	FJ613419	Sheffield et al. (2009)
	*Macrodorcas seguyi*	MF612068	Chen et al. (2018)
	*Neolucanus maximus*	MF401425	Direct submission
	*Nigidionus parryi*	KP987576	Direct submission
	*Nigidius* sp	JX412771	Direct submission
	*Odontolabis cuvera fallaciosa*	MF908524	Wang et al. (2018)
	*Prosopocoilus blanchardi*	KF364622	Kim et al. (2015)
	*Prosopocoilus confucius*	KU552119	Lin et al. (2017)
	*Prosopocoilus gracilis*	KP735805	Wu et al. (2015)
	*Pseuderhaetus sinicus*	KP987575	Direct submission
	*Prismognathus prossi*	MF614014	Jing et al. (2018)
	*Rhaetus westwoodi*	MG159815	Jing et al. (2018)
	*Serrognathus platymelus*	MF612070	Direct submission
	*Sinodendron yunnanense*	KP735804	Lin et al. (2017)
Scarabaeidae	*Cheirotonus jansoni*	NC023246	Shao et al. (2014)
	*Protaetia brevitarsis*	NC023453	Kim et al. (2014)
	*Rhopaea magnicornis*	NC013252	Cameron et al. (2009)

The stag beetle *L. cervus* is one of the species that is in a protected status in many European countries. This species has been included in Annex II of the EU Habitats Directive since 1992 (Council Directive 92/43/EEC of 21 May 1992 on the Conservation of Natural Habitats and of Wild Fauna and Flora) (Bardiani et al., 2017), and the European Union finances “Life” Projects to protect the species every year. Nevertheless, populations of this stag beetle have dramatically declined in recent years in several European countries, mainly due to fragmentation and loss of suitable habitats (Campanaro et al., 2016). *L. cervus* is the largest European beetle and is widely distributed in Europe (Harvey et al., 2011). It is noteworthy that *L. cervus* spends most of its life in larval and pupal stage; its saproxylic larva feeds in rotten wood or roots in deciduous broad-leaved forests, in lowland and medium-altitude areas (Tini et al., 2017). Previous works revealed that the species shows a similar life history across its range, but the habitat conditions in different European countries (such as climate, temperature, humidity, and food availability) significantly affects the larval life duration and also the adult body size (Harvey et al., 2011). The larvae show a marked increase in size in favorable conditions; the adult, especially in male specimens, can show strong morphological variability in size and shape (Romiti et al., 2017).

In certain areas of the S. Mediterranean region (e.g., Latium in Italy), *L. cervus* can be morphologically confused and can even have hybrids with the congeneric *L. tetraodon* (a species not included in protection lists). In some areas of Central Italy, *L. cervus* has a sympatric occurrence with the closely related species *L. tetraodon*, and individuals with a mosaic of morphological traits can be found, making the species assignment on a simple morphological basis nearly impossible. More or less similar problems can raise at the south-eastern borders of the Palearctic distribution of *L. cervus* (e.g., in Turkey or Near East) where populations of other closely related species of *Lucanus* converge (Cox et al., 2013).

In this study, we sequenced the complete mitochondrial genome of *L. cervus* using next-generation sequencing; we inferred phylogenetic relationships among 20 stag beetle species. The aim of this study is to contribute to research on phylogeny of the family Lucanidae and to provide genomic information that could be useful for better management and conservation strategies that impact on this species.

## Materials and Methods

### Sample collection and DNA extraction

The voucher specimen of *L. cervus* was collected in Ukraine in July 2017 and deposited in the Museum of Anhui University with the accession number Lu166. Total genomic DNA was extracted from the muscle of *L. cervus* using the Qiagen DNAeasy Kit. The sequence was submitted to GenBank and assigned Accession Number MN580549 and converts into graphical maps utilized Organellar Genome DRAW in the GenBank format (Greiner, Lehwark & Bock, 2019).

### Polymerase chain reaction amplification, and sequencing

Polymerase chain reaction (PCR) amplification reactions for *cox1*, *cytb*, and *rrnl* were carried out in 25 μL volumes containing 2 μL template DNA, 12.5 μL 2 × EasyTaq SuperMix (+dye), 1 μM of each primer (forward and reverse), and 8.5 μL sterile double-distilled water. Three fragments were amplified using common primers for *L. cervus* (Table 2). The PCR amplifications were performed under the conditions as previously described in Lin et al. (2017). Sanger sequencing was used to obtain the fragments of *cox1*, *cytb* and *rrnl*. A library was prepared using Truseq nano DNA kit (Illumina) with an insert size of 450 bp, and sequenced on the Illumina HiSeq 2,000 platformat Berry Genomics, Beijing, China. Raw reads were trimmed using Trimmomatic, with matched to the adaptor sequence >15 bp, poly-Ns (>15 bp Ns) or >75 bp bases with quality score ≤3 (Bolger, Lohse & Usadel, 2014) and we obtained 98,22,905 clean 250 bp paired-end reads with the Q30 = 93.25.

**Table 2:** Information of the primers used in this study.

Gene	Primer name	Sequence (5′–3′)	Reference
*cox1*	COI–F1	CAACATTTATTTTGATTTTTTGG	Simon et al. (1994)
	COI–R1	TCCAATGCACTAATCTGCCATATTA	Simon et al. (1994)
*cytb*	Cytb–F2	GAGGAGCAACTGTAATTACTAA	Balke, Ribera & Vogler (2004)
	Cytb–R2	AAAAGAAARTATCATTCAGGTTGAAT	Balke, Ribera & Vogler (2004)
*rrnl*	16S–F1	CCGGTTTGAACTCAGATCATG	Hosoya, Honda & Araya (2001)
	16S–R1	TAATTTATTGTACCTTGTGTATCAG	Hosoya, Honda & Araya (2001)

### Genome assembly, annotation and analysis

High-quality reads were de novo assembled using IDBA-UD (Peng et al., 2012) with the parameters: minimum *k* value 80, similarity threshold 98%, and maximum *k* value 240. The *cox1* (555 bp), *cytb* (404 bp) and *rrnl* (865 bp) fragments of *L. cervus* were used to identify mitochondrial assemblies using BLAST searches with ≥98% similarity (Altschul et al., 1990). To verify the accuracy of the assembly, clean reads were mapped onto the obtained fragments using “Map to reference” option of Geneious Prime 2019.1.1 (https://www.geneious.com) with maximum mismatches per read 2%; maximum ambiguity two; minimum overlap identity 97%; minimum overlap 100 bp; and no gaps. When genome was assembled in full length, the two ends of the contig overlapped, indicating circular organization of the mitochondrial genome. Finally, we obtained the mitochondrial genome of *L. cervus* with the mean coverage of 252.

The mitochondrial genome sequence was annotated using the MITOS web server (Bernt et al., 2013). tRNA genes and their secondary structures were inferred using tRNAscan-SE v2.0 (Lowe & Chan, 2016). In addition to 16S ribosomal RNA (*rrnl*, lrRNA), and 12S ribosomal RNA (*rrns*, srRNA), *trnS*1 (TCT) also determined according to sequence similarity with related species because of can’t identified by tRNAscan-SE. The codon usage, nucleotide compositions of PCGs were calculated with MEGA 7 (Kumar, Stecher & Tamura, 2016). Composition skew analysis was conducted according to formulas *AT* skew = (*A* − *T*)/(*A* + *T*) and *GC* skew = (*G* − *C*)/(*G* + *C*) (Perna & Kocher, 1995).

### Phylogenetic analysis

We retrieved 19 complete or near complete mitogenomic sequences from GenBank (Table 1) and added a newly sequenced *L. cervus* generating a dataset of 20 species. Three mitochondrial genomes from the family Scarabaeidae were used as outgroups. Twenty species represent 14 genera of Lucanidae. The lacked genes were treated as missing data in mitochondrial genomes of *Dorcus parallelipipedus* (lack of *rrnl*), *Nigidius* sp. (lack of *rrns*), and *Nigidionus parryi* (lack of *nad2*, *cox1*, *rrns*, *rrnl*). The dataset containing nucleotide sequences of 13 PCGs-condon12 with the third position removed and two rRNA genes (*rrns* and *rrnl*) of 22 species. Each PCG was aligned individually based on codon-based multiple alignments using MEGA 7 (Kumar, Stecher & Tamura, 2016). PCG12 was produced by removing the third codon with MEGA 7 (Kumar, Stecher & Tamura, 2016). Two rRNA genes (*rrnl* and *rrns*) was aligned individually using MAFFT 7 server with the G-INS-i strategy (Katoh, Rozewicki & Yamada, 2017; Kuraku et al., 2013), and we used Gblocks v0.91b Server (Castresana, 2000; Talavera & Castresana, 2007) to select conserved blocks from multiple alignments. Final, we concatenated alignments using Geneious Prime 2019.1.1 (https://www.geneious.com). The PCG12RNA dataset was partitioned using PartitionFinder 2 (Lanfear et al., 2017) for maximum likelihood (ML) analyses.

Bayesian inference (BI) and ML analyses were conducted using PhyloBayes MPI 1.5a (Lartillot, Lepage & Blanquart, 2009) and IQ-TREE web server, respectively. The BI analysis was carried out on the CIPRES Science Gateway (Miller, Pfeiffer & Schwartz, 2011). We chose the site-heterogeneous mixture model (CAT + GTR) (Song et al., 2016). Two independent chains starting from a random tree were run for 20,000 cycles, with trees being sampled every 10 cycles. The initial 25% trees of each MCMC run were discarded as burn-in. A consensus tree was computed from the remaining 1,500 trees combined from two runs, and the two runs converged at a maxdiff of less than 0.1. The best scheme for ML analyses see Table S1. For ML analyses, the “Auto” option was set under optimal evolutionary models, and the phylogenetic trees were constructed using an ultrafast bootstrap approximation approach with 10,000 replicates. Phylogenetic trees were viewed and edited in Figtree v1.4.3.

## Results

### Genome organization and base composition

**Figure 1:**
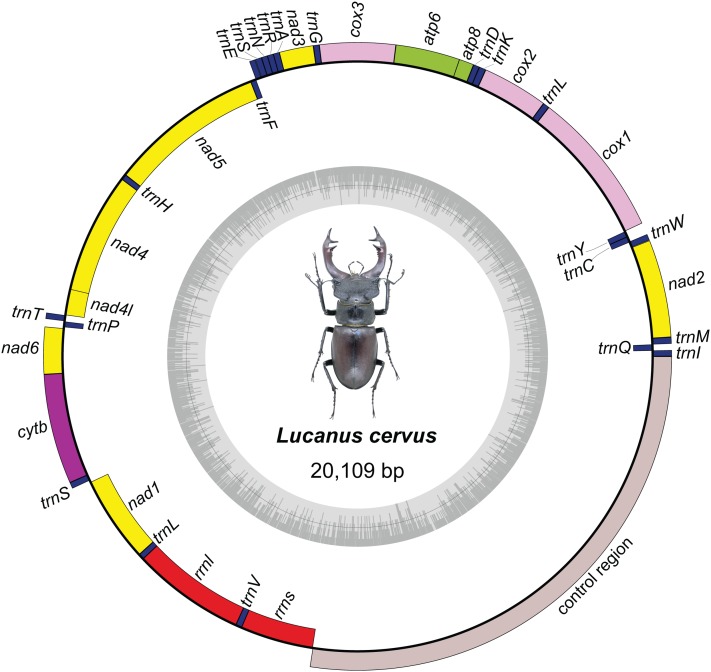
Mitochondrial genome map of *Lucanus cervus*. Different types of genes are represented by different colors in the genome map: *nad1*, *nad2*, *nad3*, *nad4*, *nad4l*, *nad5*, and *nad6* refer to nicotinamide adenine dinucleotide subunit indicated by yellow; *cox1*, *cox2*, and *cox3* refer to cytochrome oxidase subunit indicated by pink; *atp6* and *atp8* refer to ATP synthase F0 subunit indicated by green; *cytb* refers to cytochrome B indicated by purple; *rrnl* and *rrns* refers to the large and small subunit of ribosomal RNA genes respectively, and both of them indicated by red; all transfer RNA indicated by deep blue; control region indicated by gray.

The complete mitochondrial genome of *L. cervus* is 20,109 bp in length. It is a closed circular molecule (Fig. 1), consisting of 22 tRNAs, two ribosomal RNA genes (*rrnl* and *rrns*), 13 PCGs and one control region as other typical stag beetles (Lin et al., 2017; Liu et al., 2019; Wu et al., 2015). Overall the mitogenome consisted of 37.48% *A*, 32.45% *T*, 19.05% *C* and 11.02% *G* with a highly biased *A* + *T* content of 69.93% (Table 3). Compared with *L. mazama* and *L. foutunei*, *L. cervus* has higher *A* + *T* content, especially in the control region. But the *A* + *T* content of *L. cervus* is at a medium level within the Lucanidae which has the variable base composition (Chen et al., 2018). Additionally, there is a negative *GC* skew, and a positive *AT* skew of *L. cervus* as other stag beetles (Chen et al., 2018; Lin et al., 2017; Liu et al., 2019, 2017; Jing et al., 2018; Wu et al., 2015). The bias of base composition has important reference value for studying the mechanism of mitochondrial genome replication and transcription (Wei et al., 2010).

**Table 3:** Base composition of different regions of the mitochondrial genome of *Lucanus cervus*.

Region	Length (bp)	*T* (%)	*C* (%)	*A* (%)	*G* (%)	*A* + *T* (%)	*AT* skew	*GC* skew
*atp6*	668	38.62	20.36	29.49	11.53	68.11	−0.13	−0.28
*atp8*	156	37.82	19.87	35.90	6.41	73.72	−0.03	−0.51
*cox1*	1,531	34.81	20.18	29.33	15.68	64.14	−0.09	−0.13
*cox2*	684	36.40	20.61	30.70	12.28	67.11	−0.08	−0.25
*cox3*	785	35.54	21.66	28.41	14.39	63.95	−0.11	−0.20
*cytb*	1,141	36.02	22.26	29.18	12.53	65.21	−0.10	−0.28
*nad1*	951	26.18	19.87	43.43	10.52	69.61	0.25	−0.31
*nad2*	1,014	37.18	21.89	31.56	9.37	68.74	−0.08	−0.40
*nad3*	354	37.57	19.49	31.64	11.30	69.21	−0.09	−0.27
*nad4l*	1,334	25.64	19.42	45.65	9.30	71.29	0.28	−0.35
*nad4l*	288	26.74	15.28	48.96	9.03	75.69	0.29	−0.26
*nad5*	1,715	27.52	19.01	43.79	9.68	71.31	0.23	−0.33
*nad6*	498	38.35	18.47	33.94	9.24	72.29	−0.06	−0.33
*rrnl*	1,244	34.41	18.49	39.31	7.80	73.71	0.07	−0.41
*rrns*	806	35.86	17.37	38.21	8.56	74.07	0.03	−0.34
rRNAs	2,050	34.98	18.05	38.88	8.10	73.85	0.05	−0.38
tRNAs	1,426	34.22	15.5	38.78	11.5	73	0.06	−0.15
13PCGs	11,119	32.65	20.16	35.82	11.37	68.47	0.05	−0.28
Control region	5,516	30.49	18.11	40.10	11.29	70.59	0.14	−0.23
Whole genome	20,109	32.45	19.05	37.48	11.02	69.93	0.07	−0.27

### PCGs and codon usage

In PCGs, four (*nad4*, *nad4l*, *nad5*, *nad1*) of the 13 PCGs were coded on the N-strand, with the other nine genes (*cox1*, *cox2*, *cox3*, *atp8*, *atp6*, *nad2*, *nad3*, *nad6*, and *cytb*) were coded on the J-strand. Among the 13 PCGs, the longest was the *nad5* gene and the shortest was the *atp8* gene. The start codons of *cox1* are AAT whereas other 12 PCGs are ATN codons (Table 4). Seven of the 13 PCGs shared the typical termination codons TAA and TAG, while others use *TA* residue or a single *T* as the terminator codons (Table 4). It is generally accepted that incomplete codon structures signal a halt of protein translation in insects and other invertebrates (Cheng et al., 2016).

**Table 4:** Characteristics of the mitochondrial genome of *Lucanus cervus*. J and N indicates the Majority strand and Minority strand of mt genome, respectively.

Gene	Strand	Region	Length (bp)	Start codon	Stop codon	Anticodon	Intergenic nucleotides (bp)
*trnI*	J	1–64	64			GAT	0
*trnQ*	N	62–130	69			TTG	−3
*trnM*	J	130–197	68			CAT	−1
*nad2*	J	198–1,211	1,014	ATC	TAG		0
*trnW*	J	1,214–1,279	66			TCA	2
*trnC*	N	1,272–1,336	65			GCA	−8
*trnY*	N	1,337–1,400	64			GTA	0
*cox1*	J	1,402–2,932	1,531	AAT	T		1
*trnL* (CUN)	J	2,933–2,998	66			TAA	0
*cox2*	J	2,999–3,682	684	ATA	TAA		0
*trnK*	J	3,683–3,752	70			CTT	0
*trnD*	J	3,753–3,815	63			GTC	0
*atp8*	J	3,816–3,971	156	ATT	TAA		0
*atp6*	J	3,968–4,635	668	ATA	TA		−4
*cox3*	J	4,635–5,419	785	ATG	TA		−1
*trnG*	J	5,419–5,480	62			TCC	−1
*nad3*	J	5,481–5,834	354	ATT	TAG		0
*trnA*	J	5,833–5,897	65			TGC	−1
*trnR*	J	5,897–5,960	64			TCG	−1
*trnN*	J	5,960–6,023	64			GTT	−1
*trnS* (AGN)	J	6,024–6,090	67			TCT	0
*trnE*	J	6,092–6,153	62			TTC	1
*trnF*	N	6,152–6,214	63			GAA	−2
*nad5*	N	6,214–7,928	1,715	ATT	TA		−1
*trnH*	N	7,929–7,990	62			GTG	0
*nad4*	N	7,990–9,323	1,334	ATG	TA		−1
*nad4l*	N	9,317–9,604	288	ATG	TAA		−7
*trnT*	J	9,607–9,670	64			TGT	2
*trnP*	N	9,675–9,736	62			TGG	4
*nad6*	J	9,741–10,238	498	ATG	TAA		4
*cytb*	J	10,238–11,378	1,141	ATG	T		−1
*trnS* (UCN)	J	11,379–11,443	65			TGA	0
*nad1*	N	11,463–12,413	951	ATT	TAG		19
*trnL* (CUN)	N	12,414–12,476	63			TAG	0
*rrnl*	N	12,477–13,720	1,244				0
*trnV*	N	13,721–13,788	68			TAC	−8
*rrns*	N	13,788–14,593	806				−1
Control region	N	14,594–20,109	5,516				0

### tRNA and rRNA genes

All of the 22 tRNAs had a total length of 1,426 bp and range from 61 to 71 bp (Table 4), eight of the 22 tRNA-coding genes were located on the N-strand and others were located on the J-strand. Secondary structures predicted by the tRNA scan-SE suggested that all the tRNA genes in *L. cervus* adopted a typical clover-leaf structure, expect for *trnS1* (TCT) is absent due to the deficiency of the dihydrouridine arm, which is a typical feature of metazoan mitochondrial genomes (Cameron, 2014). The length of *rrnl* and *rrns* were 1,252 bp and 806 bp, respectively (Table 4).

### Control region

The control region of *L. cervus* was located between *trnI* and *rrns* genes as normally with the length of 5516 bp; The *A* + *T* contents, *AT*-skew and *GC*-skew is 70.59%, 0.14 and −0.23, respectively. There were seven poly-*T* (≥7) stretch, 24 poly-*A* (≥7) stretch, and one poly-*TA* (≥7) sequence in the long control region. Furthermore, two tandem repeats were found by online Tandem repeats finder (Benson, 1999), both of which were repeated three times; the length of the consensus sequence was 272 bp, 278 bp, respectively.

### Phylogenetic analysis

**Figure 2:**
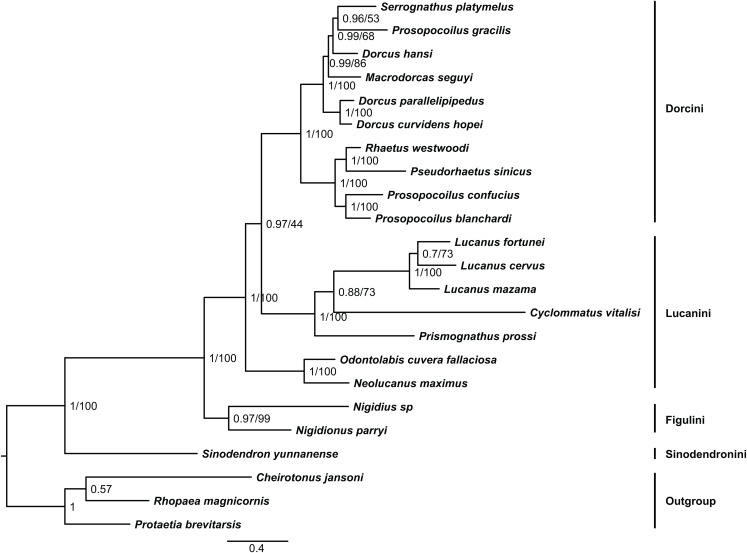
Combination of the BI and ML. Phylogenetic relationships among the Lucanidae inferred from Bayesian inference and Maximum Likelihood methods based on PCG12RNA dataset. We selected 19 stag beetles as ingroup and three other scarab species (*Rhopaea magnicornis*, *Protaetia brevitarsis* and *Cheirotonus jansoni*) as outgroup. These two methods obtained consistent topologies in the ingroup. The line with 0.4 is a scale for scaled branches of BI and shows the evolutionary distance of each species. Values near the the node show the posterior probability of both methods of corresponding node.

Including the newly sequenced mitochondrial genome of *L. cervus*, a total of twenty mitochondrial genomes from Lucanidae were used for phylogenetic analysis to analyze their phylogenetic relationships. Both BI and ML analyses consistently showed phylogenetic relationships among Lucanidae (Fig. 2). *Sinodendron yunnanense* is an early branch in Lucanidae (BPP = 1, MLB = 100). Lucanini was not monophyletically supported, and the earliest branch lineage was comprised of genera *Neolucanus* and *Odontolabis*. *Prismognathus* and *Cyclommatus* were closely related to *Lucanus*, they clustered into a lineage and sister to Dorcini. Additionally, *L. cervus* and *L. fortunei* clustered into a lineage (BPP = 0.7, MLB = 73) and sister to *L. mazama* (BPP = 1, MLB = 100).

## Discussion

The control region is the most important non-coding region in the mitochondrial genome with extremely abundant *A* + *T* content, and the length variation is very large (Zhang & Hewitt, 1997; Zhang, Szymura & Hewitt, 1995), even among species having a close genetic relationship. *L. cervus* has a control region with the length of 5,516 bp, obviously longer than other Lucanini species reported in our previous work (Chen et al., 2018; Lin et al., 2017; Liu et al., 2019, 2017; Jing et al., 2018; Wu et al., 2015), causing its total mitochondrial genome to be significantly longer than others.

This study presents consistent phylogenetic relationships basing BI and ML methods. Overall, there was no apparent effect of long-branch attraction within the ingroup. Most of our findings based on BI and ML were congruent with the phylogenetic analyses based on a combination of several mitochondrial genes and nuclear ribosomal genes (Kim et al., 2015). However, there were controversial relationships, especially in the lower taxonomic levels. For example, the phylogenetic status of genera *Neolucanus* and *Odontolabis* in our results were different from previous results generated from multiple fragments (Kim et al., 2015). *Prosopocoilus gracilis* has been classified into *Dorcus* (s.l.) rather than *Prosopocoilus* (Lin et al., 2017). Our results showed that mitochondrial genomes sequences are powerful in relationships inference within Lucanidae. The sequencing and assembly of the mitochondrial genome will facilitate future works of mitochondrial genome sequencing (Bourguignon et al., 2017; Crampton-Platt et al., 2015). Increased taxa sampling and genome sequencing will help to resolve the classification problems within Lucanidae.

## Conclusions

In our study, the phylogenomic analysis supports the morphological conclusion on relationships of Lucanidae. Although our data could not solve all the phylogenetic relationships within Lucanidae, this study can be helpful for future research on the Lucanidae phylogeny.

## Supplemental Information

10.7717/peerj.8274/supp-1 Supplemental Information 1 Reference cited in Table 1 and Table 2.

10.7717/peerj.8274/supp-2 Supplemental Information 2 Annotation information for MN580549.

10.7717/peerj.8274/supp-3 Supplemental Information 3 The sequence of *Lucanus cervus*.

10.7717/peerj.8274/supp-4 Supplemental Information 4 Partition for IQ-TREE identified by PartitionFinder.

## Additional Information and Declarations

### DNA Deposition

The following information was supplied regarding the deposition of DNA sequences: The voucher specimen of *Lucanus cervus* is deposited in the Museum of Anhui University (Lu166) and is also available in GenBank: MN580549.

### Data Availability

The following information was supplied regarding data availability: The raw measurements are available in the Supplemental Files.
