# Supplementary material for: The complete mitochondrial genome of stag beetle Lucanus cervus (Coleoptera: Lucanidae) and phylogenetic analysis
Source: PeerJ. 2019 Dec 19;7:e8274. doi: 10.7717/peerj.8274 (PMC6925956; doi:10.7717/peerj.8274)
Supplement: Supplemental Information 1 [file peerj-07-8274-s001.doc]

**Reference in Table 1**

Cameron SL, Sullivan J, Song HJ, Miller KB, and Whiting MF. 2009. A mitochondrial genome phylogeny of the Neuropterida (lace-wings, alderflies and snakeflies) and their relationship to the other holometabolous insect orders. *Zoologica Scripta* 38:575-590.

Chen YJ, Liu J, Cao Y, Zhou S, and Wan X. 2018. Two new complete mitochondrial genomes of Dorcus stag beetles (Coleoptera, Lucanidae). *Genes Genomics* 40:873-880. DOI 10.1007/s13258-018-0699-8.

Kim MJ, Im HH, Lee KY, Han YS, and Kim I. 2014. Complete mitochondrial genome of the whiter-spotted flower chafer, *Protaetia brevitarsis* (Coleoptera: Scarabaeidae). *Mitochondrial DNA* 25:177-178. DOI 10.3109/19401736.2013.792064.

Kim MJ, Kim K-G, Kim SR, and Kim I. 2015. Complete mitochondrial genome of the two-spotted stag beetle, *Metopodontus blanchardi* (Coleoptera: Lucanidae). *Mitochondrial DNA* 26:307-309. DOI 10.3109/19401736.2013.825788.

Linard B, Arribas P, Andújar C, Crampton-Platt A, and Vogler AP. 2016. Lessons from genome skimming of arthropod-preserving ethanol. *Molecular Ecology Resources* 16:1365-1377. 10.1111/1755-0998.12539.

Liu J, Li CG, You S, and Wan X. 2017. The first complete mitogenome of *Cyclommatus* stag beetles (Coleoptera: Lucanidae) with the phylogenetic implications. *Entomotaxonomia* 39:294-299. DOI 10.11680/entomotax.2017035.

Liu J, Cao Y, Zhou S, Chen Y, and Wan X. 2019. Complete mitochondrial genome of *Prismognathus prossi* (Coleoptera: Lucanidae) with phylogenetic implications. *Entomologica Fennica* 30:1-7.

Liu J, Zhou SJ, Chen YJ, and Wan X. 2018. Mitogenome of the Monotypic Genus *Rhaetus* (Coleoptera: Scarabaeidae: Lucanidae). *Journal of entomological science* 53:503-513.

Lin ZQ, Song F, Li T, Wu YY, and Wan X. 2017. New Mitogenomes of Two Chinese Stag Beetles (Coleoptera, Lucanidae) and Their Implications for Systematics. *Journal of Insect Science* 17. DOI 10.1093/jisesa/iex041.

Shao LL, Huang DY, Sun XY, Hao JS, Cheng CH, Zhang W, and Yang Q. 2014. Complete mitochondrial genome sequence of *Cheirotonus jansoni* (Coleoptera: Scarabaeidae). *Genetics and molecular research* 13:1047-1058. 10.4238/2014.February.20.6.

Sheffield NC, Song H, Cameron SL, and Whiting MF. 2009. Nonstationary evolution and compositional heterogeneity in beetle mitochondrial phylogenomics. *Systematic Biology* 58:381-394. 10.1093/sysbio/syp037.

Wang Q, Liu J, Lin ZQ, and Wan X. 2018. The complete mitochondrial genome of *Odontolabis fallaciosa*（Coleoptera：Lucanidae）with its phylogenetic implications. *Zoological Systematics* 3.

Wu YY, Cao YY, Fang J, and Wan X. 2015. The first complete mitochondrial genome of stag beetle from China, *Prosopocoilus gracilis* (Coleoptera, Lucanidae). *Mitochondrial DNA* 27:2633-2634. DOI 10.3109/19401736.2015.1041129.

**Reference in Table 2**

Balke M, Ribera I, and Vogler AP. 2004. MtDNA phylogeny and biogeography of Copelatinae, a highly diverse group of tropical diving beetles (Dytiscidae). *Molecular Phylogenetics and Evolution* 32:866–880.

Hosoya T, M. Honda, and K. Araya. 2001. Genetic variations of 16S rRNA gene observed in Ceruchus lignarius and *Dorcus rectus rectus* (Coleoptera: Lucanidae). *Journal of entomological science* 4:335–344.

Simon C, Frati F, Beckenbach A, Crespi B, Liu H, and Flook P. 1994. Evolution, Weighting, and Phylogenetic Utility of Mitochondrial Gene Sequences and a Compilation of Conserved Polymerase Chain Reaction Primers | Annals of the Entomological Society of America | Oxford Academic. *Annals of the Entomological Society of America* 87:651-701.
