# Supplementary material for: The complete mitochondrial genome of stag beetle Lucanus cervus (Coleoptera: Lucanidae) and phylogenetic analysis
Source: PeerJ. 2019 Dec 19;7:e8274. doi: 10.7717/peerj.8274 (PMC6925956; doi:10.7717/peerj.8274)
Supplement: Supplemental Information 4 [file peerj-07-8274-s004.docx]

| Subset | Partition |
| --- | --- |
| 1 | cob_pos1, atp6_pos1 |
| 2 | cob_pos2, atp6_pos2, nad3_pos2 |
| 3 | nad6_pos1, atp8_pos1 |
| 4 | nad2_pos2, nad6_pos2, atp8_pos2 |
| 5 | cox1_pos1, cox3_pos1 |
| 6 | cox1_pos2, cox3_pos2 |
| 7 | cox2_pos2, cox2_pos1 |
| 8 | nad4l_pos1, nad5_pos1, nad4_pos1 |
| 9 | nad1_pos2, nad4l_pos2, nad5_pos2, nad4_pos2 |
| 10 | nad3_pos1, nad2_pos1 |
| 11 | rrns |
| 12 | rrnl |
